# The Role of Sleep in Retention of New Words in Habitually and Non-Habitually Napping Children

**DOI:** 10.3390/brainsci11101320

**Published:** 2021-10-05

**Authors:** Katherine Esterline, Rebecca L. Gómez

**Affiliations:** Psychology Department, The University of Arizona, Tucson, AZ 85721, USA; rgomez@arizona.edu

**Keywords:** word learning, sleep, memory, napping, development

## Abstract

Daytime napping contributes to retention of new word learning in children. Importantly, children transition out of regular napping between ages 3–5 years, and the impact of this transition on memory is unclear. Here, we examined the performance of both non-habitually napping children (nap 0–3 days per week, *n* = 28) and habitually napping children (nap 4–7 days per week, *n* = 30) on a word learning task after a delay including either sleep or wakefulness. Children ages 3.5–4.5 years old experienced a brief exposure to two novel labels and their referents during training, a scenario that replicates learning experiences children encounter every day. After a 4-h delay, children were tested on the object-label associations. Using mixed effects logistic regression, we compared retention performance. Non-habitual nappers and habitual nappers displayed a different pattern of retention such that non-habitually napping children did equally well on a test of retention regardless of whether they napped or stayed awake during the delay. In contrast, habitually napping children needed a nap after learning to retain the novel object-label associations 4 h later. As a group, habitual nappers who remained awake after learning performed no better than chance on the retention test. As children transition out of naps, they may be less susceptible to interference and are better able to retain newly learned words across a delay including wakefulness.

## 1. Introduction

By the age of two years, most children are acquiring new vocabulary at a rapidly increasing rate, an important development given the central role of vocabulary in communication. Perry and Horst [1] point to three crucial phases inherent in learning a new word: (1) forming an association between a label and a meaning or referent, (2) remembering the association over a delay, and (3) applying the new mapping to a novel situation [1]. We examine the second phase of word learning in the work presented here.

### 1.1. Assessing Memory via Single Exposure Word Learning across a Delay

Though most of the word learning literature has focused on children’s performance at encoding [2,3], examining children’s retention across delays provides insight into memory processes and factors that influence acquiring new vocabulary. Thus, the current study employs a word learning paradigm to examine memory for new words across a delay. Additionally, most word learning studies present children with multiple exposures to a given object–label pairing. However, neural circuitry is maturing, suggesting that by preschool-age, children may be able to encode and retain novel words after minimal exposure [4,5]. Furthermore, in many real-life learning situations, children may only be exposed to a novel word and its referent for a brief amount of time. Although a recent study examined word learning after a brief learning event, children in the study were given three exposures to the words and were tested 30 min after learning [6]. Thus, the question of how children will learn after only a single exposure to novel words, and how this representation will endure over longer delays, remains unexplored.

### 1.2. Napping Regularity and Learning

Previous work has demonstrated that daytime sleep contributes to memory consolidation—an important consideration for vocabulary acquisition—in infants [7,8,9,10,11,12] and children [13,14,15,16,17]. An additional consideration in the relationship between daytime sleep and memory in children is that daytime sleep patterns across a 24-h period change substantially across early development and naps are eventually phased out. Early in childhood, children take 1 or 2 naps during the day [18], but gradually the duration of daytime sleep decreases, and between ages 3–5 years, children begin to forgo naps entirely and sleep only at night [19,20,21].

A few studies have explored how the transition out of napping impacts learning and memory, but questions remain. In one study, 3-year-old children aged 35 to 42 months were classified as either a habitual napper (naps 4–7 days per week) or a non-habitual napper (naps 0–3 days per week). From these groups, children were then randomly assigned to “Nap” or “Wakefulness” groups. In the nap groups, children napped soon after learning pairings of novel verb labels with novel actions. In the Wakefulness groups, children remained awake after the learning session. Children were then tested on their ability to generalize the novel verbs to new actors performing the action 24 h later. Children who napped after learning generalized, whereas children who remained awake after learning did not. This study showed that young 3-year-olds benefit from naps regardless of their typical napping status [15].

Similarly, another study with a slightly older age group showed that non-habitually napping children may be missing out on the memory benefit of a nap. In this study, 3.5-year-old children were exposed to novel words via shared storybook readings. Children were either read the same storybook featuring the new words throughout the learning phase, or they were exposed to the new words via different storybooks. In this design, children who were non-habitual nappers stayed awake after learning and children who were habitual nappers took their nap after learning. Children who napped after learning (i.e., habitual nappers) outperformed children who remained awake (i.e., non-habitual nappers). Critically, when non-habitual nappers were exposed to the novel words via different storybooks (a more cognitively demanding task [22]), they never reached the performance of the other children at follow-up tests [17]. Thus, these authors also demonstrate that non-habitually napping children (who stay awake after learning) do not perform as well as habitually napping children who nap after learning.

A third study shows a slightly different pattern of results. Kurdziel et al. [14] taught preschool-aged children (ages 3–5.5 years) the spatial locations of objects on a grid and, after learning, children napped or stayed awake. Children’s memory for these object–location associations was tested soon after the nap or a period of wakefulness and 24-h later. When children napped, they retained more than when they stayed awake. Though, when considering the child’s typical napping behavior, the authors showed that this effect was especially true in children who habitually napped 5 days or more per week, suggesting that the benefit of the nap is most pronounced in habitually napping children. Importantly, children who had transitioned out of regular napping maintained accuracy for object–location associations across a period of wakefulness compared to habitual nappers, who showed greater memory decay across wakefulness [14].

Taken together, Sandoval et al. [15] and Williams and Horst [17] suggest that children who have transitioned out of naps do not retain novel information across periods of wakefulness as well as their habitually napping peers. However, Kurdziel et al. [14] show that non-habitually napping children maintain accuracy across periods of wakefulness compared to habitual nappers. What might account for these different findings in response to wakefulness? Age may be a factor. Importantly, in the two word learning studies described above [15,17] children were on the younger side (3-years-old and 3.5-years-old, respectively) of the typical age range for nap cessation—around 3–5 years old [19,20,21]. Perhaps these younger non-habitual nappers show different patterns in response to periods of wakefulness than children who transition out of naps later. Thus, examining how habitual napping behavior impacts novel word learning across a wider and older age range of 3.5–4.5 years may show a more representative pattern of the interaction between naps, wakefulness, and learning.

Better understanding the transition out of napping and how it impacts learning could have important implications for educational settings and public policy. If preschools and daycares remove or reduce napping from children’s schedules, this could result in some children missing naps that they would still benefit from that may have negative effects on their learning. For example, if a young child would otherwise nap regularly, but is not able to nap at school, what effect might this have on their retention of new learning? Clarifying the role of a child’s typical napping behavior on the relationship between sleep and learning may help in determining when children should cease napping.

The goal of the current study is to examine the retention of new word learning in habitually napping and non-habitually napping children after a delay including either sleep or wakefulness. We hope to clarify the role of children’s typical napping behavior in sleep and learning and what this can tell us about when children should cease napping. Children were first classified as a habitual napper or a non-habitual napper based on their typical napping behavior. During the training sessions, children were given a brief exposure to two novel labels and their referents—a scenario that may be a more stringent test of retention of new words after a delay. This brief exposure also replicates learning experiences children encounter every day where they might experience one brief instance of a novel object-label pairing. After training, half of the children took their typical nap, and half of the children stayed awake. Four hours later, children were tested on their ability to select the target object between the two previously labeled objects in a two-alternative forced choice task.

According to Contextual Binding theory, memories encoded soon before sleep are subject to less contextual interference as sleep reduces the possibility of encoding new information that would share a similar context as the learning experience [23]. However, this theory does not take development into account and does not predict different outcomes as a function of developmental maturity. Previous studies suggest that children who nap regularly may have less mature memory networks compared to children who no longer nap regularly [14,24]. If so, children who have transitioned (or are transitioning) out of naps should better retain new information across periods of wakefulness, and thus will exhibit successful retention regardless of whether they nap or stay awake during the delay. We predict that children who nap regularly will need to nap soon after learning in order to retain the novel associations 4 h later.

## 2. Materials and Methods

### 2.1. Participants

Fifty-eight children (29 females) aged 4 years (M = 47.34 months, range = 41.56–53.95 months) participated in this study. We recruited children aged 3.5–4.5 years in order to sample equal numbers of children who nap habitually and no longer nap habitually, as children transition out of naps between ages 3 and 5 years [19,21,25]. We also elected to keep the recruitment range wide (1 year) as we wanted to obtain data from children transitioning out of naps at different ages throughout that window.

**Figure 1:**
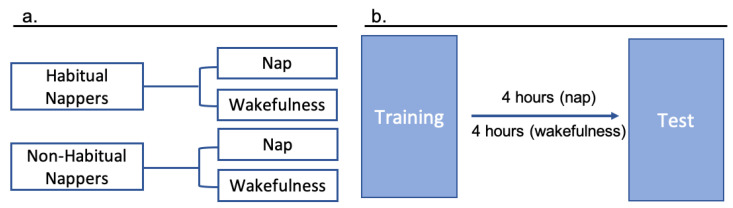
Study Design. Children were first categorized as a “Habitual Napper” or a “Non-Habitual Napper” and were then randomly assigned to either “Nap” or “Wakefulness” groups (**a**). All children participated in a training session, where they learned the names of novel toys and had a testing session 4 h later (**b**). Children in the nap groups napped between training and test and children in the wakefulness groups remained awake during the delay.

Children were first assigned to “Habitual Napper” or “Non-Habitual Napper” groups based on their napping patterns reported by their parents. Children were considered habitual nappers if they napped 4–7 days per week and non-habitual nappers if they napped 0–3 days per week [15]. Within these groups, children were then randomly assigned to nap-after-learning (“Nap”) groups or remain-awake-after-learning (“Wakefulness”) groups, resulting in four experimental groups: Habitual Nappers/Nap (*n* = 16; M = 48.39 months; range = 42.36–53.95 months), Habitual Nappers/Wakefulness (*n* = 14; M = 45.36 months; range = 42.03–50.69 months), Non-Habitual Nappers/Nap (*n* = 8; M = 47.93 months; range = 42.46–53.2 months), and Non-Habitual Nappers/Wakefulness (*n* = 20; M = 47.69 months; range = 41.56–53.62 months) (Figure 1a). See Table 1.

An additional 9 children were tested, but their data was discarded due to inattentiveness during the training or test session (*n* = 2), not completing the second session of the study (*n* = 1), the test session occurring more than 4 h after training (*n* = 1), receiving more than one exposure to the novel label (parent repeated labels; *n* = 1), child’s nap taking place in the car on the way to the laboratory (*n* = 3), or child’s nap lasting less than 30 min (*n* = 1). Further, 11 children were tested but their data was not included in the analyses due to condition deviations; 2 children in the Habitual Nappers/Nap group who did not nap after learning, 1 child in the Habitual Nappers/Wakefulness group who ended up napping after learning, 5 children in the Non-Habitual Nappers/Nap group who were not able to nap after learning, and 3 children in the Non-Habitual Nappers/Wakefulness group who ended up napping after learning.

A sample size of 20 children per group was estimated using G*Power calculations [26]. Values from a representative paper [15] were used in the calculation. Data collection for this study was halted due to the coronavirus pandemic, thus the target n for all groups was not obtained, though 73% of the target sample was collected.

### 2.2. Design

All children received a training session where they were taught the names of novel toys and a test session where they were tested on the toys’ names. For all children, the test session took place 4 h after the training session (Figure 1b). In the Nap conditions (Habitual Nappers/Nap and Non-Habitual Nappers/Nap), the training sessions were scheduled approximately 30 min to 1 h before the child’s typical nap time. For children in this group who were transitioning out of naps, the sessions were scheduled on a day when the child typically napped. For children in this group who had transitioned out of naps entirely, parents were asked to estimate when their child was most likely to nap, and the training session was scheduled approximately 30 min to 1 h before the child was likely to nap. In the Wakefulness conditions (Habitual Nappers/Wakefulness and Non-Habitual Nappers/Wakefulness), the training session was scheduled at a time when the child was expected to be awake until the testing session 4 h later. Thus, children in the Wakefulness conditions were not deprived of their nap, they were simply scheduled at a time when they were expected to be awake for at least 4 h. In order to verify adherence to the assigned sleep or wakefulness condition a subset of children wore an Actiwatch (Actiwatch 2, Philips, Andover, MA, USA) during the delay (Habitual Nappers/Nap, *n* = 8; Habitual Nappers/Wakefulness, *n* = 6; Non-Habitual Nappers/Nap, *n* = 4; and Non-Habitual Nappers/Wakefulness, *n* = 5). An Actiwatch is a device that measures motion and sleep-wakefulness activity. Of the children who wore the Actiwatch, all displayed activity consistent with their assigned experimental condition (Nap or Wakefulness in the 4-h window after training). Additionally, Actiwatch data showed that all children in the Nap condition met the 30-min minimum nap criterion during the post-training 4-h window.

### 2.3. Materials

For the word learning task, novel objects were created from craft supplies and differed in color and distinguishing features. Each object was given a novel label (bame, gart, baf, or fum). Additionally, the Peabody Picture Vocabulary Test [27] was administered as a measure of the child’s receptive vocabulary and estimate of intellectual ability, as well as to identify possible differences in habitual and non-habitual nappers on this measure. Parents also filled out a questionnaire on their child’s sleep patterns. The sleep questionnaire included questions about their child’s typical bedtime and risetime, number of naps their child takes per day, number of nap days per week, and, if their child had transitioned or begun to transition out of napping, the reason for transition out of napping (e.g., preschool/daycare schedule, child’s desire to transition out of napping, activities that interfere with naptime, or decision from caregivers). Parents also completed the subscales of the Social Functioning/Atypical Behaviors domain of the Conners Early Childhood Questionnaire [28] to assess whether habitual and non-habitual nappers scored differently on behavioral and developmental milestones. The Connors Early Child Questionnaire assesses children on 19 domains: Inattention/Hyperactivity, Defiant/Aggressive Behaviors, Defiance/Temper, Aggression, Social Functioning/Atypical Behaviors, Social Functioning, Atypical Behaviors, Anxiety, Mood and Affect, Physical Symptoms, Sleep Problems, Global Index: Restless-Impulsive, Global Index: Emotional Lability, Global Index: Total, Adaptive Skills, Communication, Motor Skills, Play, and Pre-Academic/Cognitive. Children also wore an Actiwatch and parents filled out a sleep diary for a week prior to the day of the study.

### 2.4. Procedure

All children participated in a training session and a test session 4 h later. Children learned two novel object-label pairings during the training session and were tested on their memory for the pairings during the test session. Half of the children napped after learning and the other half remained awake. A nap was defined as at least 30 min of sleep activity during the 4 h delay [15,16].

**Figure 2:**
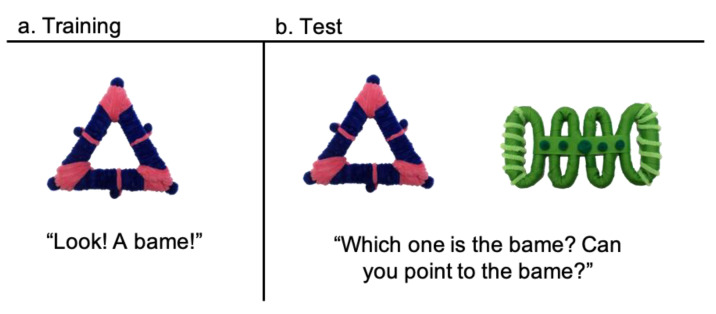
Training and Test Procedures. During training (**a**), children received a single, brief exposure to 2 novel objects and labels. A testing session occurred 4 h later (**b**) where children were given a two-alternative forced choice test between the newly learned objects.

Training: The training session took place in participants’ homes in a quiet area away from distractions. Children were told that they were going to play a game where they would learn about new toys. Children sat on the floor across from the experimenter. They received a single exposure to each of two novel object-label pairs. During presentation, the experimenter pulled the object from a toy box and placed it in front of the child. She then labeled the object once (e.g., “Look! A bame!”) and placed it back in the toy box (Figure 2a). The same procedure followed for the second novel object. Importantly, children had only one exposure to each novel object. The time of training was scheduled before the child’s typical nap for nap groups or at a time where the child was expected to be awake for at least 4 h for wakefulness groups (see “Section 2.2 Design”).

Test: The testing session took place in the laboratory 4 h after training. During this delay, children either napped or stayed awake (during a time they would typically nap or be awake, see “Section 2.2 Design”). At this session, children were tested on their memory for the object-label associations. Similar to the training session, children sat on the floor across from the experimenter. Children first participated in a pointing assessment with two familiar objects to ensure that each child understood how to use pointing to indicate their choices in the task. All children passed this assessment. Children then received four trials of a two-alternative forced choice test between the two newly learned objects (Figure 2b). For each trial, the experimenter pulled the two objects from the toy box and placed them side-by-side in front of the child. The experimenter then asked the child to point to the target object (e.g., “Which one is the bame? Can you point to the bame?”). The side the target object appeared on was counterbalanced across trials. After the word learning testing session, children participated in the PPVT. Parents received the Conners Early Childhood Questionnaire at the beginning of the training session and completed it by the end of the testing session.

## 3. Results

**Figure 3:**
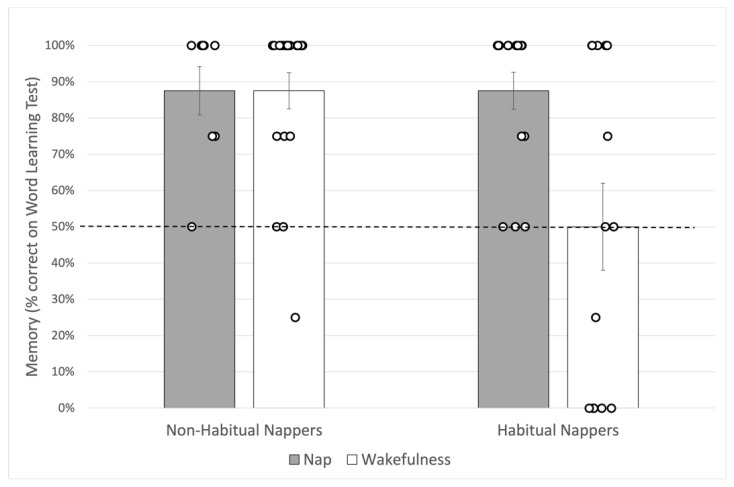
Average Memory Performance by Napping Status and Delay Type. Memory performance score averaged across four test trials. Error bars reflect standard error. Chance performance is 50%.

Averaged memory performance for each group in our experimental design is shown in Figure 3.

### 3.1. Interaction between Napping Status and Sleep versus Wakefulness during Delay

In order to determine whether typical napping behavior plays a role in the relationship between memory and sleep after a delay including a nap or wakefulness, we conducted a mixed effects regression [29] predicting the log odds of correctly choosing the target object at test as a function of delay type (Nap or Wakefulness) and typical napping status (Habitual Napper or Non-Habitual Napper). We allowed delay type and nap status to interact in order to determine whether memory performance after a period of sleep or wakefulness would differ between habitual and non-habitual nappers. The outcome variable was the score (correct or incorrect) for each of four test trials. Table 2 displays the results of this regression. First, napping status was a significant predictor of memory performance after a delay such that habitual napping was associated with lower odds of correctly selecting the target object at test. The interaction between delay type and typical napping behavior did not significantly predict memory performance after a delay, *p* = 0.057, though trended towards significance. Importantly, as discussed above, we were not able to achieve equal group sizes due to a halt in data collection related to the coronavirus pandemic. Thus, with equal sample sizes, significance may have been achieved.

### 3.2. Differences in Outcomes Based on Nap Habituality

As previous studies have suggested developmental differences in memory networks between habitually napping children and non-habitually napping children [14,24], we divided our sample based on typical napping status and analyzed each group separately to determine any differences in memory performance in response to a period of sleep or wakefulness after learning.

We conducted a second mixed effects regression for habitually napping children and used delay type (Nap or Wakefulness) as a predictor. The results of this analysis are displayed in Table 3. Here, we found that delay type did predict memory performance after a 4-h delay such that the odds of selecting the correct target object at test for habitually napping children who nap after learning are 59.5 times that of habitually napping children who remain awake after learning.

Finally, we conducted a third mixed effects regression for non-habitually napping children and used delay type (Nap or Wakefulness) as a predictor. The results of this analysis are displayed in Table 4. Here, delay type did not significantly predict the log odds of selecting the target object at test, suggesting a period of sleep or wakefulness after learning does not impact memory performance for non-habitually napping children.

In order to determine whether children remembered the novel objects significantly better than would be expected by chance, trial scores were averaged for each child and compared to chance performance (50%) by experimental group, using one-tailed Wilcoxon signed rank tests. Only habitually napping children who remained awake after learning failed to perform significantly better than chance, *p* > 0.05. All other groups exceeded chance performance: Habitual Nappers/Nap, *p* < 0.001, *r* = 0.88; Non-Habitual Nappers/Wakefulness, *p* < 0.001, *r* = 0.86; and, despite the low group size (*n* = 8), Non-habitual Nappers/Nap, *p* = 0.009, *r* = 0.89 with power = 78%. As the effect size for the comparison to 50% chance in Habitual Nappers/wakefulness was exactly zero, an estimated effect size of 0.001 with the desired power of 80% would require a sample size of 6,182,559 children to detect an effect, a sample size we would not achieve, pandemic or not.

### 3.3. Control Variables, Standardized Measures, and Patterns of Sleep

To explore the possibility that any memory differences between habitual nappers and non-habitual nappers may stem from differences in vocabulary, we conducted an independent sample *t* test on children’s standard PPVT scores (see Table 5). This revealed no differences between habitual nappers (*M* = 114.5) and non-habitual nappers (*M* = 111.8); *t*(48) = 0.78, *p* > 0.05, *d* = 0.22. Additionally, we compared habitual and non-habitual nappers on the 19 subscales of the Conners Early Childhood Questionnaire and found no differences between children based on habitual napping behavior (all *p*s > 0.05). Further, we confirmed no differences in age between habitual nappers (*M* = 47.0) and non-habitual nappers (*M* = 47.8), *t*(56) = −0.78, *p* > 0.05, *d* = −0.20.

Data from the 1-week sleep diary were also analyzed to confirm that the number of naps per week as reported in the sleep diary was consistent with the initial condition assignment. Complete sleep diary data was obtained from 36 children (Habitual Nappers/Nap: 10, Habitual Nappers/Wakefulness: 8, Non-Habitual Nappers/Nap: 7, and Non-Habitual Nappers/Wakefulness: 11). Though a few of the sleep diary reports were off by 2 or fewer days compared to the initial parent reported napping days, all were consistent with the condition assignment of Habitual Napper (4–7 days per week) and Non-Habitual Napper (0–3 days per week).

As training and test times were scheduled in 4 h intervals around children’s typical nap time (nap groups) or a time when the child would typically be awake (wakefulness groups), we expected to see differences in time of training and time of test between the nap and wakefulness groups. Indeed, among Habitual Nappers, children in the wakefulness group were trained and tested at a significantly earlier time of day (*M_Train_ =* 9:29 a.m. and *M_Test_* = 1:31 p.m.) compared to children in the sleep group (*M_Train_* = 12:30 p.m. and *M_Test_* = 4:30 p.m.), *t*(14) = 4.03, *p* < 0.001, *d* = 1.56 for time of training and *t*(16) = 4.02, *p* < 0.001, *d* = 1.55 for time of test. Similarly, among Non-Habitual Nappers, children in the wakefulness group were trained at a significantly earlier time of day (*M_Train_ =* 9:58 a.m. and *M_Test_* = 1:58 p.m.) compared to children in the sleep group (*M_Train_* = 12:22 p.m. and *M_Test_* = 4:26 p.m.), *t*(25) = 5.96, *p* < 0.001, *d* = 1.72 for time of training and *t*(25) = 6.17, *p* < 0.001, *d* = 1.77 for time of test. Importantly though, there was not a significant difference between children in the Habitual Nap/Wakefulness and Non-Habitual Nap/Wakefulness groups in time of training, *t*(20) = −0.61, *p* = 0.57, *d* = −0.23, or test, *t*(20) = −0.61, *p* = 0.55, *d* = −0.22, suggesting that the time of day training and test occurred is not related to the differences in retention we see here.

We also examined the wakefulness interval (i.e., the amount of time between last sleep and training) and found a significant difference between children in the Nap conditions (*M* = 5.6 h) and children in the Wakefulness conditions (*M* = 2.3 h), *t*(54) = 8.79, *p* < 0.001, *d* = 2.15.

For non-habitually napping children, parents selected the reasons their child transitioned out of naps from the following: preschool/daycare schedule, child’s desire to transition out of napping, activities that interfere with naptime, or decision from caregivers. Parents could select multiple options. The vast majority of parents indicated that at least one of the reasons for nap cessation was their child’s desire to stop napping (83%). In addition, 24% of parents reported that activities interfered with their child’s nap time, 14% of parents indicated that attending preschool or daycare prompted the transition out of naps, and 17% of parents indicated that it was a decision by the caregivers.

## 4. Discussion

Daytime sleep has previously been shown to impact retention of new word learning in early childhood [8,9,10,13,15,17], yet children transition out of regular napping, so the question of how this transition impacts sleep and memory in children remains unclear. Here, we examine the impact of typical napping patterns on retention of new words after a delay including either sleep or wakefulness. Children were given a single exposure to two novel words, then either took their typical nap or stayed awake during the four hours after training. Children were then tested on the object–label pairings in a two-alternative forced choice task. Here, we found a different pattern of retention based on napping status. Non-habitually napping children (those who have mostly or fully transitioned out of regular napping) remember the object–label pairings after a 4-h delay regardless of whether they nap after learning or not. Children who still nap regularly show much more variability in their responses. On average such children must take a nap after learning in order to remember the object-label pairings 4 h later. Importantly, this study also demonstrates that children are able to retain novel object–label pairings after only a brief exposure (one labeling of a novel word mapping).

These findings may suggest that as children transition out of naps, they are better able to retain novel information across longer periods of wakefulness. Interestingly, our behavioral findings map onto imaging results comparing regularly napping children with children who had transitioned out of naps. Children who rarely nap have smaller hippocampal subfield volumes [30], consistent with findings that indicate a reduction in hippocampal subfield volume with maturation [31]. Children who rarely napped also showed better memory on an ordered recall task than children of the same age who were still napping occasionally or regularly, leading the authors to propose that children who no longer nap regularly have more developed memory networks [30].

Our behavioral results discussed here also complement neurophysiological data showing documented changes in slow-wave activity across early childhood. Slow-wave activity (SWA), a type of slow oscillatory electroencephalography (EEG) activity, has long been considered a marker of sleep need: SWA increases across wakefulness and decreases across sleep. Kurth et al. [32] measured children’s naps at three different ages using electroencephalography (EEG). At each age, children’s naps were measured at three times across the day—in the morning, afternoon, and evening. Among the developmental changes in nap physiology, a notable finding is a decrease in slow-wave activity (SWA) in children’s naps as they mature. SWA has long been considered a physiological marker of sleep pressure [33], so these findings suggest that as children age, they accumulate sleep pressure at a slower rate, allowing them to remain awake for longer periods of time, and eventually phase out napping. As such, were we to add polysomnography to our design, we might expect to see higher SWA in the naps of habitually napping children that should correlate with retention compared to those who no longer nap habitually.

Importantly, we observe a different pattern of results than two previous studies that also examined children’s napping status and word learning. Sandoval et al. [15] demonstrated that, regardless of napping status, young 3-year-olds generalized novel verb learning when they had a nap soon after learning—but not when they remained awake after learning. Similarly, Williams and Horst [17] showed that non-habitually napping 3.5-year-olds who stayed awake after learning novel words did not retain as much of their learning as habitually napping children who had napped after learning. In the current study, we purposely recruited children from a slightly older age range (42–53 months, *M* = 47 months, or just under 4-years-old). Thus, is it possible that children who transition out of napping earlier in life may show a different response to periods of wakefulness after learning than children who transition out of naps slightly later.

In the current study, a notable finding is that children in the Wakefulness groups showed a striking difference in memory performance across a 4-h period of wakefulness based on their typical napping status. Though the same age on average, habitually napping children who stayed awake after learning performed much worse on average (no better than chance) on the memory task compared to non-habitually napping children who stayed awake after learning. Importantly, these children were not deprived of a nap—they were simply scheduled during a time they would already be awake. This finding may suggest that, as children transition out of naps, they become less susceptible to interference across periods of wakefulness (see Riggins and Spencer [24] for a similar argument). Indeed, evidence from both human and animal studies show that memory representations are susceptible to interference during waking and are resistant to interference once sleep is initiated [34]. More mature memory networks [24] may make non-habitual nappers more resistant to interference during wakefulness compared to habitual nappers.

A recent theory on memory formation and retention, the contextual binding (CB) theory, proposes that sleep benefits memory, not through active consolidation, but through a reduction in contextual interference [23]. In this theory, the hippocampus binds together contextual elements of an experience (for example, spatial, temporal, cognitive aspects, etc.) and according to CB, gradual changes in context result in memories that share similar context, which causes interference and forgetting. In this view, memories encoded soon before sleep are subject to less contextual interference, as sleep reduces the possibility of encoding new information that would share a similar context as the learning experience. The results of the current study can be interpreted within this framework as we see that, regardless of typical napping behavior, children who sleep soon after learning retain the object–label associations. However, this model would also predict that children who remain awake after learning would be subject to contextual interference as representations for experiences they encounter soon after learning would share some similar context. Importantly, we only see this pattern in children who nap habitually—non-habitually napping children who remain awake after learning retain the object–label associations, even after a period of wakefulness. Considered within the framework of CB, perhaps children who have transitioned out of naps are less susceptible to contextual interference than typical nappers. If children who are still napping regularly have less mature memory networks, as suggested by Riggins and Spencer [24], perhaps they have less distinct contextual representations that lead to greater contextual overlap between memories and greater forgetting.

Another possibility is that non-habitual nappers may be more cognitively mature and therefore any differences in retention between non-habitual nappers and habitual nappers may be attributed to cognitive differences between the two. However, unpublished work in our laboratory using this paradigm showed equal performance for habitual and non-habitual nappers on an immediate test (habitual nappers, *M* = 0.69, *SD* = 0.37; non-habitual nappers, *M* = 0.73, *SD* = 0.35, *t*(46) *=* −0.45, *p* > 0.05, *d* = −0.13), suggesting equal encoding regardless of napping status. Additionally, in the current study, we found no evidence for vocabulary differences between habitual and non-habitual nappers. This finding is consistent with a previous study that found no difference in PPVT scores between non-habitually napping and habitually napping 3-year-olds [15]. These findings are inconsistent with Lam et al. [35] who showed that smaller vocabularies were associated with more napping during the week. However, as discussed in Sandoval et al. [15], differences in nighttime sleep duration may play help explain this difference. Children in the Lam et al. [35] study obtained a lower amount of nighttime sleep than is recommended for that age group [36]. Therefore, if children are obtaining too little nighttime sleep, compensatory napping during the day may interfere with their nighttime sleep in either duration or quality, which may in turn impact cognitive functioning.

We did find a significant difference in the wakefulness interval between when children last slept and training such that children in the Nap groups, overall, experienced a longer waking interval before training than did children in the Wakefulness groups. This finding is not surprising, given the study design and scheduling restrictions based on children’s typical sleep and wakefulness patterns. However, one concern could be that a shorter wakefulness interval for the wakefulness group might result in better encoding and hence better retention than for the nap group. If this were the case, we would expect to see the best performance in the wakefulness groups (Non-Habitual Nappers/Wakefulness and Habitual Nappers/Wakefulness) and the worst performance in the nap groups (Non-Habitual Nappers/Nap and Habitual Nappers/Nap), but this is not what we observe. In fact, we see excellent performance in both Nap groups, and we see the worst performance in the Habitual-Napper/Wakefulness group. Furthermore, if we compare wakefulness intervals across napping status there are no differences (Non-Habitual Nappers, *M* = 3.63 h and Habitual Nappers, *M* = 3.69 h). If we look just within the Wakefulness groups, Non-Habitual Nappers averaged 2.86 h of wakefulness before training and Habitual Nappers averaged 1.56 h of wakefulness before training, and yet this latter group showed the worst performance. Thus, it does not seem that a short wakefulness interval before training aids performance.

Additionally, as expected, we did find that children in the wakefulness groups were trained and tested earlier than children in the nap groups. This is not surprising, as children’s naps tend to occur mid-day, and the wakefulness groups’ training and test were scheduled around nap times. Importantly, however, there were no differences in training and test times between Habitual Nappers and Non-Habitual Nappers who remained awake after training, which is where we observed a difference in retention. This suggests that circadian differences during training and test cannot explain differences in retention between these two groups.

The current study has a few limitations. First, most parents of non-habitual nappers in this study indicated that their child transitioned out of regular napping of their own desire. This may indicate that children in the current study transitioned out of naps as they were ready, rather than due to external pressures. It is possible that the pattern we found here may reflect the relationship between sleep and memory across a natural nap transition, and children who transition out of naps due to external factors may show a different pattern of response to a period of wakefulness.

Second, as indicated above, we scheduled children at times of day that accorded with their natural sleep and wakefulness schedules. Thus, we trained and tested children in sleep and wakefulness groups at different times of day. However, circadian effects did not contribute to our main finding as habitual and nonhabitual nappers in the wakefulness groups trained and tested at the same times of day.

Additionally, the current study cannot discern whether the pattern of results found could be attributed to active processes during sleep or the nap offering protection from interference. One previous study has shown that spindle activity in children’s naps correlates positively with their change in memory performance on an object–location memory task, suggesting sleep played an active role in consolidation [14]. However, we did not employ polysomnography in the current study. Future studies could measure nap physiology after novel word learning in children to address this. Furthermore, future studies should specifically measure the impact of interference during a period of wakefulness in non-habitual nappers compared to habitual nappers. If non-habitual nappers are less impacted by an interference task after novel learning compared to habitual nappers, as would be predicted from the findings in our current study, it would be evidence that, as children transition out of naps, they become less susceptible to interference allowing them to retain novel information across longer periods of wakefulness.

Finally, due to the coronavirus pandemic, data collection was halted, so complete samples could not be obtained. With a complete sample, we may have been able to better detect an interaction between habitual napping status and delay type. Furthermore, the size of our Non-Habitual Nappers/Nap group is relatively small (*n* = 8). We anticipated this problem, given that once children have transitioned out of regular napping, it may be difficult for them to nap during the day—a fact that is reflected in the number of children who were originally assigned to this group but were unable to nap during the delay (*n* = 5). However, a power analysis was conducted for this group showing that, despite the low group size, sufficient power (78%) was achieved. Additionally, our best estimate of effect size for our Habitual Nappers/Wakefulness group requires a sample size of 6,182,599 children to obtain significance, underscoring the fact that our target N would not result in significance for this group.

## 5. Conclusions

We show that children who have transitioned out of regular napping retain novel words after a 4-h delay, regardless of whether they napped after learning or not. However, children who still regularly nap must take a nap soon after learning in order to retain the novel object–label associations. When habitual nappers remained awake after learning, they performed no better than chance at test on average. Importantly, this effect is not purely age-related, as there were no differences in average age between habitual and non-habitual nappers. More research is needed to better understand if daytime naps play an active versus protective role (or both) on memories in early childhood. The current study is consistent with the literature in showing that sleep is important for memory consolidation in children and adds new findings demonstrating that a child’s regular napping behavior may play a role in the relationship between sleep and memory.

## Figures and Tables

**Table 1:** Sample Composition by Group.

	Habitual Nappers/Nap	Habitual Nappers/Wake	Non-Habitual Nappers/Nap	Non-Habitual Nappers/Wake	*p*
n male (%) female (%)	16 9 (56%) 7 (44%)	14 7 (50%) 7 (50%)	8 3 (37.5%) 5 (62.5%)	20 10 (50%) 10 (50%)	
Average age in months (SD)	48.39 (3.84)	45.36 (3.12)	47.93 (3.68)	47.69 (4.0)	ns

**Table 2:** Fixed Effect Estimates for Mixed Effects Model of Memory Performance as a Function of Delay Type and Nap Status.

Fixed Effect	*ß*	*SE*	Wald *z*	*p*
Intercept	3.485	0.975	3.574	<0.001
Nap Status	−3.485	1.256	−2.776	<0.01
Delay	−0.194	1.349	−0.144	0.886
Nap Status × Delay	3.623	1.901	1.906	0.057

*ß* reflects estimates of the coefficients associated with the effect, *SE* is an estimate of the standard error of the coefficients, and Wald *z* represents the coefficient estimates’ distance from zero—in terms of the standard error.

**Table 3:** Fixed Effect Estimates for Mixed Effects Model of Memory Performance as a Function of Delay Type in Habitual Nappers.

Fixed Effect	*ß*	*SE*	Wald *z*	*p*
Intercept	<0.001	1.013	0.00	1.00
Delay	4.086	1.785	2.290	<0.05

**Table 4:** Fixed Effect Estimates for Mixed Effects Model of Memory Performance as a Function of Delay Type in Non-Habitual Nappers.

Fixed Effect	*ß*	*SE*	Wald *z*	*p*
Intercept	2.738	0.797	3.437	<0.001
Delay	−0.089	1.005	−0.088	0.930

**Table 5:** Means and Standard Deviations for Habitual Nappers and Non-Habitual Nappers.

	Habitual Nappers	Non-Habitual Nappers	*t*
Age (months)	47.0 (3.79)	47.8 (3.84)	ns
Typical Nighttime Sleep (hrs)	11.0 (3.3)	10.8 (0.8)	ns
Total Sleep (hrs)	12.8 (3.3)	11.6 (1.3)	ns
Naps Per Week	5.4 (1.1)	0.8 (1.1)	15.04 ***
PPVT Score	114.5 (11.9)	111.8 (12.8)	ns

Standard deviations in parentheses, *** *p* < 0.001.

## References

[1] Perry L., Horst J. (2019). Learning, Recognizing, and Extending the Meanings Words. International Handbook of Language Acquisition.

[2] Vlach H.A. (2019). Learning to Remember Words: Memory Constraints as Double-Edged Sword Mechanisms of Language Development. Child Dev. Perspect..

[3] Wojcik E.H. (2013). Remembering New Words: Integrating Early Memory Development into Word Learning. Front. Psychol..

[4] Gómez R.L., Edgin J.O. (2016). The Extended Trajectory of Hippocampal Development: Implications for Early Memory Development and Disorder. Dev. Cogn. Neurosci..

[5] Lee J.K., Nordahl C.W., Amaral D.G., Lee A., Solomon M., Ghetti S. (2015). Assessing hippocampal development and language in early childhood: Evidence from a new application of the Automatic Segmentation Adapter Tool. Hum. Brain Mapp..

[6] Remon D., Loevenbruck H., Deudon M., Girardie O., Bouyer K., Pascalis O., Thorpe S. (2020). 24-Month-Olds and over Remember Novel Object Names after a Single Learning Event. J. Exp. Child Psychol..

[7] Gómez R.L., Bootzin R.R., Nadel L. (2006). Naps Promote Abstraction in Language-Learning Infants. Psychol. Sci..

[8] Horváth K., Myers K., Foster R., Plunkett K. (2015). Napping Facilitates Word Learning in Early Lexical Development. J. Sleep Res..

[9] Horváth K., Liu S., Plunkett K. (2016). A Daytime Nap Facilitates Generalization of Word Meanings in Young Toddlers. Sleep.

[10] Horváth K., Plunkett K. (2016). Frequent Daytime Naps Predict Vocabulary Growth in Early Childhood. J. Child Psychol. Psychiatr..

[11] Hupbach A., Gomez R.L., Bootzin R.R., Nadel L. (2009). Nap-Dependent Learning in Infants: Nap-Dependent Learning in Infants. Dev. Sci..

[12] Seehagen S., Konrad C., Herbert J.S., Schneider S. (2015). Timely Sleep Facilitates Declarative Memory Consolidation in Infants. Proc. Natl. Acad. Sci. USA.

[13] Axelsson E.L., Swinton J., Winiger A.I., Horst J.S. (2018). Napping and Toddlers’ Memory for Fast-Mapped Words. First Lang..

[14] Kurdziel L., Duclos K., Spencer R.M.C. (2013). Sleep Spindles in Midday Naps Enhance Learning in Preschool Children. Proc. Natl. Acad. Sci. USA.

[15] Sandoval M., Leclerc J.A., Gómez R.L. (2017). Words to Sleep On: Naps Facilitate Verb Generalization in Habitually and Nonhabitually Napping Preschoolers. Child Dev..

[16] Werchan D.M., Kim J.-S., Gómez R.L. (2021). A Daytime Nap Combined with Nighttime Sleep Promotes Learning in Toddlers. J. Exp. Child Psychol..

[17] Williams S.E., Horst J.S. (2014). Goodnight Book: Sleep Consolidation Improves Word Learning via Storybooks. Front. Psychol..

[18] Mindell J.A., Owens J.A., Carskadon M.A. (1999). Developmental Features of Sleep. Child Adolesc. Psychiatr. Clin. N. Am..

[19] Davis K.F., Parker K.P., Montgomery G.L. (2004). Sleep in Infants and Young Children. J. Pediatric Health Care.

[20] Staton S., Rankin P.S., Harding M., Smith S.S., Westwood E., LeBourgeois M.K., Thorpe K.J. (2020). Many Naps, One Nap, None: A Systematic Review and Meta-Analysis of Napping Patterns in Children 0–12 Years. Sleep Med. Rev..

[21] Weissbluth M. (1995). Naps in Children: 6 Months–7 Years. Sleep.

[22] Horst J.S. (2013). Context and Repetition in Word Learning. Front. Psychol..

[23] Yonelinas A.P., Ranganath C., Ekstrom A.D., Wiltgen B.J. (2019). A Contextual Binding Theory of Episodic Memory: Systems Consolidation Reconsidered. Nat. Rev. Neurosci..

[24] Riggins T., Spencer R.M.C. (2020). Habitual Sleep Is Associated with Both Source Memory and Hippocampal Subfield Volume during Early Childhood. Sci. Rep..

[25] Iglowstein I., Jenni O.G., Molinari L., Largo R.H. (2003). Sleep Duration from Infancy to Adolescence: Reference Values and Generational Trends. Pediatrics.

[26] Faul F., Erdfelder E., Lang A.-G., Buchner A. (2007). G*Power 3: A Flexible Statistical Power Analysis Program for the Social, Behavioral, and Biomedical Sciences. Behav. Res. Methods.

[27] Dunn L., Dunn D. (2007). PPVT-4: Peabody Picture Vocabulary Test.

[28] Conners C. (2009). Conners Early Childhood. Multi-Health Syst..

[29] Jaeger T.F. (2008). Categorical data analysis: Away from ANOVAs (transformation or not) and towards logit mixed models. J. Mem. Lang..

[30] Riggins T., Spencer R.M.C. To Nap or Not: Relations between Napping, Brain Development, and Memory in Preschool Children. Proceedings of the Society for Research in Child Development.

[31] Riggins T., Geng F., Botdorf M., Canada K., Cox L., Hancock G.R. (2018). Protracted Hippocampal Development Is Associated with Age-Related Improvements in Memory during Early Childhood. NeuroImage.

[32] Kurth S., Lassonde J.M., Pierpoint L.A., Rusterholz T., Jenni O.G., McClain I.J., Achermann P., LeBourgeois M.K. (2016). Development of Nap Neurophysiology: Preliminary Insights into Sleep Regulation in Early Childhood. J. Sleep Res..

[33] Rasch B., Born J. (2013). About Sleep’s Role in Memory. Physiol. Rev..

[34] Seibt J., Frank M.G. (2019). Primed to Sleep: The Dynamics of Synaptic Plasticity Across Brain States. Front. Syst. Neurosci..

[35] Lam J.C., Mahone E.M., Mason T., Scharf S.M. (2011). The Effects of Napping on Cognitive Function in Preschoolers. J. Dev. Behav. Pediatrics.

[36] Mindell J., Owens J. (2015). A Clinical Guide to Pediatric Sleep: Diagnosis and Management of Sleep Problems.

